# Anaemia control and the interpretation of biochemical tests for iron status in children

**DOI:** 10.1186/s13104-017-2472-5

**Published:** 2017-04-26

**Authors:** Thando P. Gwetu, Meera K. Chhagan, Myra Taylor, Shuaib Kauchali, Murray Craib

**Affiliations:** 1 Discipline of Public Health Medicine, University of KwaZulu-Natal, Private Bag X7, Durban, 4013 South Africa; 2 Department of Paediatrics and Child Health, University of KwaZulu-Natal, Durban, South Africa

**Keywords:** Anaemia, Iron deficiency, Inflammation, Ferritin, Serum transferrin receptor, Body iron, Parasite infection

## Abstract

**Background:**

Anaemia is one of the world’s most prevalent child health problems. Its control in Africa and other developing nations has been hindered by uncertainty regarding its cause. Anaemia control has been particularly problematic in regions where the non-iron deficiency causes of anaemia, are projected to be substantial. The implementation of effective interventions to reduce the anaemia prevalence, requires improved documentation on iron status and other causes of anaemia for target populations.

**Methods:**

This cross-sectional study enrolled n = 184 children, aged 6–8 years from Kwazulu-Natal, South Africa. Tests of haemoglobin, serum ferritin, soluble transferrin receptor and C-reactive protein were performed. These conventional measures of iron status were used to calculate body iron and to categorize the children into different groups of anaemia profiles.

**Results:**

Anaemia prevalence was high, 43/184 (23.4%). Iron deficiency anaemia contributed 7/43 (16.3%) to the anaemia prevalence compared to non-iron deficiency anaemia 34/43 (79.1%) and mixed anaemia 2/43 (4.7%). In total 47/184 (25.5%) of the sampled children had either iron deficiency or anaemia. Information about the presence of inflammation was used to adjust serum ferritin concentrations, resulting in improved diagnosis of iron deficiency.

**Conclusion:**

Appropriate investigations for iron status and inflammation/infection screening, need to be integral in the evaluation of anaemia and its causes before anaemia control interventions are implemented. Interventions that target the multifactorial nature of anaemia in school-aged children need to be strengthened. Additionally, regular screening of anaemia in school-aged children from disadvantaged communities is recommended.

## Background

The prevalence of anaemia in low-income nations is estimated to be three to four times higher than that of industrialized states [1]. However, few studies in developing countries have reported on the burden of factors associated with anaemia in children at national or community levels. Information on the various factors contributing to the anaemia burden is essential when designing interventions to reduce the impact of anaemia. The high prevalence of anaemia in developing countries persists, probably because interventions are often conducted assuming that the anaemia is due to dietary iron deficiency. Various studies have however shown that other factors such as bacterial, viral and parasitic infections, as well as various micronutrient deficiencies and other pathologies play a major role in the cause of anaemia in low and middle-income settings [2, 3] and that these factors may also affect body iron status [4]. The presence of infectious causes of anaemia may limit the response to iron supplementation, [5] and could even have negative consequences as seen in the Pemba iron and folic acid supplementation trial which was conducted in a high malaria transmission setting [6]. Some of the children sampled in this present study had received interventions for anaemia in the past. The interventions which were given to the children were assessed for their contribution to the aetiology of the present anaemia.

Conventional biochemical investigations which use serum ferritin (SF), C reactive protein (CRP) and soluble transferrin receptor (sTfR) levels for iron status and inflammation assist in the evaluation of the nature of anaemia, although there is no internationally agreed method of measuring population iron status [7]. Laboratory assessments for the analysis of iron status are generally not specific with regards to defining iron deficiency anaemia (IDA) and non-iron deficiency anaemia (NIDA). IDA occurs when haemoglobin (Hb) in the blood is decreased due to insufficient iron, resulting in the blood lacking adequate healthy red blood cells to carry oxygen to the body’s tissues. In contrast, NIDA is characterized by adequate tissue iron which is not accessible to bone marrow erythroid precursors and accompanies infections, inflammation or neoplastic conditions. The measure of sTfR and SF has been suggested as a possible source of important information in distinguishing between IDA and NIDA [8]. Serum ferritin, which is often used as an indicator of body iron stores, has a problematic interpretation in the presence of inflammation because it is also an acute phase reactant [9]. CRP was used to measure acute inflammation for this study [10]. Some researchers have however reported that SF in children may be affected by inflammation to the same extent or more than CRP [11]. The values of SF may therefore be increased despite reduced body iron levels. When the assessments for iron status were conducted, data on inflammation was used to adjust ferritin levels where sub-clinical inflammation was identified [11]. The sTfR level which is an early marker of functional iron deficiency, is generally believed to be unaffected by inflammation although some researchers have reported that its levels may be increased in the presence of infections [12]. Of note, is that sTfR measurements vary with different commercial assays due to the absence of an international standard. To increase reliability in the estimation of body iron stores, the use of a ratio of the transferrin receptor and ferritin has been recommended [7]. Both SF and sTfR go through characteristic sequential changes, as body iron stores decrease from normal iron-replete levels to IDA [7]. In the iron depletion phase (stage I), sTfR levels remain stable, though SF levels decrease. The deficit in stored iron results in reduced functional iron compounds and Hb synthesis and then iron-deficient erythropoiesis (IDE) follows (stage II). In early IDE, compensatory elevated sTfR levels are seen. IDA (stage III) occurs when Hb concentrations are lower than normal due to progressive depletion of the functional iron. Because SF represents the stored iron and sTfR reflects the functional tissue iron, a combination of these two values into a ratio is believed to be a good indicator of body iron status [7]. However, the calculation technique or the most suitable equation, for these ratios is not yet settled and continues to be a subject of debate [7, 8, 11, 12].

This present ancillary study sought to validate results obtained during phase one of the Asenze study, which identified a high anaemia burden of 53% among the sampled children when the children were aged 4–6 [13]. The Asenze study which means “let us act/do” in the local Zulu language, was in phase two of operations during this study’s data collection. The children were being followed-up after school entry at ages 6–8 years. This study expects to contribute to the discussion on anaemia control strategies in disadvantaged populations by describing anaemia aetiology among children from a deprived community and comparing the use of locally available combined biochemical indices against available traditional measures which use either SF/CRP or sTfR values for the assessment of iron status.

## Methods

All children who came to the Asenze study’s research clinic over the data collection period had an equal and independent chance of participating. This was done so as to obtain an unbiased representation of the child population attending the clinic.

### Diagnostic testing

The children’s anaemia and iron status was assessed using readily available laboratory assays for Hb, SF, sTfR and CRP. Anaemia was defined using Hb levels and was classified as mild, moderate and severe (Table 1) [14]. Low levels of SF < 15 μg/l were used to indicate iron depletion [7]. Information on inflammation was used to adjust SF concentrations using a modified method published by Thurnham et al. [11]. The samples were categorised based on CRP levels and the children were placed into two groups; the ‘apparently healthy’ who had CRP ≤ 5 mg/l and the group with inflammation/infection who had CRP > 5 mg/l. The SF values were multiplied by the ratio of the median of the ‘apparently healthy’ group to the median of the inflammation group in order to obtain the adjusted SF. The laboratory recommended sTfR level of >4.6 mg/l was used as an alternative to ferritin in identifying iron deficiency [15, 16]. CRP was used as a general marker for inflammation and infection, and hence as a very rough proxy for NIDA. CRP values ≥5 mg/l were used to identify inflammation or sub-clinical infection while values >10 mg/l were used to indicate infection [14, 17]. The estimation of the body iron (mg/kg body weight) was based on the ratio of sTfR to inflammation-adjusted SF as outlined by Cook et al. in the equation: body iron (mg/kg) = −[log10 (sTfR/SF) − 2.8229]/0.1207 [15]. The sTfR measurements from this study were validated against the Cook model [7]. The total body iron stores (mg) were calculated by multiplying body iron with body weight. The log sTfR/SF and the sTfR/log ferritin (TfR-F Index) which both combined sTfR and ferritin levels were calculated. The cut-off values for the outcome measures were as follows: (a) anaemia: Hb < 115 g/l, [14] (b) ID: body iron stores <0 mg/kg [15]; and (c) inflammation: CRP ≥ 5 mg/l [11]. These measures were used to differentiate between IDA and NIDA. Participants were classified into five groups according to anaemia, iron and inflammatory status; IDA, NIDA, iron deficient stores (IDS), mixed anaemia (MA) and non-anaemic non-iron deficient (NA) as shown in Table 1.

**Table 1:** Definition and operationalization of different groups according to anaemia and/or iron status

Category	Groups	Definition
Anaemia severity	Mild	Hb levels 110–114 g/l
	Moderate	Hb levels 80–109 g/l
	Severe	Hb < 80 g/l
Iron and anaemia status groups	IDA	Anaemia and low body iron stores in the absence of inflammation
	NIDA	Anaemia in the presence of inflammation in a child with normal iron stores
	IDS	Depleted iron stores but child was not anaemic
	MA	Anaemia in the presence of both iron deficiency and inflammation
	NA	Normal haemoglobin concentrations and normal iron status

Weight was measured according to standard procedures. Weight measurements were read to the nearest 0.1 kg on a portable Philips^®^ digital bathroom scale-model HF340/00. Two readings were undertaken for each child for accuracy. Malaria status was not assessed or accounted for, because the study area was in a non-malaria endemic region.

### Stool and urine examination

Urine and stool samples were collected to determine the relationship of anaemia and children’s parasite prevalence. Stool specimens were analysed for helminth infection through the use of the formalin–ether concentration method. Urine was analysed for Schistosoma infection which was confirmed by direct microscopy for ova. Parasite infection was further sub-divided into pathogenic and non-pathogenic parasites. Pathogenic parasites are associated with clinical symptoms such as diarrhoea, malnutrition, stunting, and cognitive impairment [18, 19]. Non-pathogenic parasites are linked with faecal–oral contamination and were considered as an indicator of poor hygiene. Parasites which were considered to be pathologic included *Ascaris lumbricoides*, *Giardia lamblia*, *Blastocystis hominis*, *Enterobius vermicularis*, *Schistosoma haematobium, Taenia and Entamoeba histolytica*. The infection which was considered as non-pathological was *Entamoeba coli.*

### Previous treatments received

In this present study the laboratory analyser was used to assess Hb levels. The HaemoCue device was used to measure the children’s haemoglobin levels during phase one of the Asenze study, and children with Hb levels <100 g/l in the earlier study were referred to their local health centres for further clinical management. A history of receiving clinical treatment for anaemia from the local health centre was obtained from caregivers during individual interviews. Caregivers were also asked to recall if the child had received any deworming treatment in the period up to one year prior to the current study.

### Statistical analyses

SPSS version 22 software package was used for data entry and analysis. The distribution of anaemia, iron deficiency and inflammation was assessed using descriptive statistics and graphical plots. Characteristics of the children in each biochemically defined group were compared using Student’s *t* test, Chi square tests and ANOVA as appropriate. Logistic regression analysis was utilised to examine relationships between iron biomarkers and different variables such as CRP and parasitic detection. All p values <0.05 were considered to be statistically significant. Accuracy pertaining to agreement between two methods of clinical measurement was reported as a Kappa statistic which gives a quantitative measure of the magnitude of agreement between two or more methods [20]. A Kappa of one shows perfect agreement, while zero reveals agreement equivalent to chance. The calculation of Kappa was based on the difference between the size of agreement present (“observed” agreement) compared to the agreement expected to be due to chance alone (“expected” agreement) [19].

## Results

### Characteristics of the study population

Among the 184 children approached, there were no refusals to participate in this study. There was a predominance of males with 109/184 (59.2%) males: 75/184 (40.8%) females. The children sampled were all apparently healthy, and did not report any recent illnesses. None of the enrolled children were symptomatic for anaemia. No statistically significant differences between the genders were identified.

The prevalence of anaemia (Hb < 115 g/l) in the sampled children was high; 43/184 (23.4%). The mean Hb level for the study sample was 122 ± 11.9 g/l with a range of 78–160 g/l. Mild and moderate forms of anaemia were the most prevalent morbidity states, accounting for 22/43 (51.2%) and 18/43 (41.9%) respectively. Severe anaemia was rare 1/43 (2.3%). Storage iron depletion was identified in 7/43 (16.3%) of the anaemic children. The presence of iron deficiency was associated with more severe forms of anaemia. Among the children with IDA 5/7 (71.4%) had moderate to severe anaemia, while among those with NIDA 12/34 (35.3%) had moderate to severe anaemia. Overall 47/184 (25.5%) of the sampled children had either iron deficiency or anaemia.

### Clinical factors

Raised CRP levels >5 mg/l were identified in 30/184 (16%) of the children. Among the children with identified inflammation, 7/30 (23.3%) had CRP levels indicating infection and 14/43 (32.6%) were anaemic. CRP levels tended to increase with ferritin levels (r = 0.06; p = 0.008) and sTfR (r = 0.083; p = 0.01), but no relationship was observed with body iron (r = 0.006; p = 0.201). The relationship of clinical factors with anaemia is shown in Table 2. A comparative analysis of the anaemia status in the present study and that of the children at baseline in phase one of the Asenze study was discussed in a separate publication [21]. The relationship between anaemia status and the children’s anthropometric measures has also been discussed in a separate paper [22].

**Table 2:** The relationship of clinical factors with anaemia

Anaemia status	Inflammation (n = 30)	Parasitic infection (n = 31)	Previously dewormed (n = 107)	Previously treated for anaemia (n = 27)
Anaemic	14/30 (46.7%)	6/31 (19.4%)	26/107 (24.3%)	18/27 (66.7%)
Non-anaemic	16/30 (53.3%)	25/31 (80.6%)	81/107 (75.7%)	9/27 (33.3%)
	p = 0.005	p = 0.03	p = 0.54	p = 0.59

### Parasitic infection

**Fig. 1:**
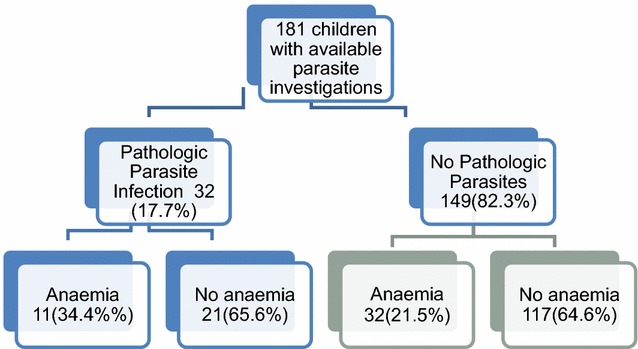
Profile of children with anaemia and parasite infection

Urine and stool samples were available for 181/184 children. Figure 1 shows the profile of the children with anaemia and parasite infection. Infection with two or more pathogenic organisms was identified in 4/181 (2.2%) children. Among those with pathogenic infection, 11/31 (35.5%) had *A. lumbricoides*, 18/31 (54.8%) *G. lamblia*, 1/31 (3.2%) *B. hominis*, 2/31 (6.5%) *E. vermicularis* and 3/31 (9.4%) *S. haematobium*. None of the children had *Taenia* infestation or *E. histolytica*. Among the children with pathogenic parasitic infections, 6/31 (19.4%) had raised CRP and 11/31 (35.5%) were anaemic. This was higher than the anaemia prevalence of 11/49 (22.4%) for all children with positive microscopy including ‘non-pathogenic’ infections. Among the participants without parasitic infection, anaemia was identified in 31/147 (21.1%) children. Variable trends for the presence of anaemia were identified among children with different parasites. The children’s anaemia prevalence in the presence of separate parasites was 4/11 (36.4%) for *A. lumbricoides* infection, 1/3 (33.3%) *S. haematobium*, 1/17 (5.9%) *G. lamblia*, 2/7 (28.6%) *Entamoeba coli* and 0/1 for *B. hominis*. Some organisms such as *E. coli* which are often classified as non-pathogens were strongly associated with anaemia (28.6%), while some pathogenic organisms such as *G. lamblia* had a weak association (5.9%). The contribution of parasite infestation to anaemia was statistically significant (p = 0.032).

### HIV infection

Caregiver consent for HIV testing was obtained for 180/184 (97.8%) children. HIV tests were positive in 5/180 (2.8%) children. Anaemia was identified in 3/5 (60%) of the HIV positive children and 1/5 (20.0%) had iron deficient stores. HIV infection resulted in a trend towards lower Hb levels (110.22 ± 8.1) g/l, compared to HIV negative children (121.9 ± 11.9) g/l. The differences between HIV-infected children were not extensively explored due to the small sample size.

### Previous clinical treatments received

During phase one of the Asenze study, 27/184 (14.7%) of the children sampled received clinical interventions for anaemia after the HaemoCue device showed Hb levels <100 g/l. Two years later, anaemia persisted in 18/27 (66.7%) of the treated children although improving Hb levels were noted using the laboratory analyser. Among the children who had received prior treatment 7/27 (25.9%) currently had Hb < 100 g/l measured by the laboratory analyser. The present anaemia profile of the children who received treatment was predominantly NIDA 14/27 (51.9%), IDA 3/27 (11.1%), MA 1/27 (3.7%) and 9/27 (33%) had iron deficiency without anaemia (IDS).

Although 107/184 (58.2%) children had received routine deworming medicine within 12 months prior to this study, no significant difference in the prevalence of anaemia was identified between the dewormed group and the children who did not receive any treatment (p = 0.54). IDS was identified in 4/107 (3.7%) children who were previously dewormed. Among the previously dewormed children 26/107 (24.3%) tested positive for current parasitic infection. The infection rate among the children who received deworming treatments, was lower than that for those who had not received any deworming treatments 27/77 (35.1%). The previously dewormed children who were anaemic during the present study had NIDA 22/26 (84.6%), IDA 3/26 (11.5%) and MA 1/26 (3.8%).

All five children who tested positive for HIV had been referred for clinical follow-up in phase one of the Asenze study. One of these children had severe anaemia at age four to six years with Hb 73 g/l and persisted to be anaemic at age 6–8 years with Hb 80 g/l. This child was referred for further investigation.

### Estimated body iron

The mean (SD) of Hb concentration was 122 ± 12 g/l, SF median was 41.5 μg/l (IQR 32.0–56.75) and sTfR median was 3.99 mg/l (IQR 3.45–4.72) Body iron averaged 0.068  ±  0.025 mg/kg, indicating that most of the children had low levels of tissue iron. Body iron stores were distributed fairly wide in this study population, resulting in a broad range (−1.722 to 3.88 mg/kg). Calculated values of body iron and body iron stores were normally distributed and were 6.8  ±  2.5 and 155 ± 66 mg/kg, respectively. Very low tissue iron was identified in 13/184 (7.1%) children.

Different methods which use the available biochemical measures were comparatively used to assess iron status, and to categorise the children into five target groups for anaemia control as shown in Tables 3 and 4. The number of children falling within each category varied according to the method used (Table 3). All five outlined methods were considered as distinct assessments for comparative analysis, and hence no specific number of children was assigned to each group.

**Table 3:** Differences in iron status and anaemia classification profiles of the sampled children according to different commonly used output measures

Definition used	Groups (n = 184)				
	IDS (%)	IDA (%)	NIDA (%)	MA (%)	NA (%)
SF/CRP	3 (1.6%)	6 (3.3%)	35 (19.0%)	2 (1.1%)	138 (75%)
sTfR	14 (7.6%)	15 (8.2%)	23 (12.5%)	5 (2.7%)	127 (69.0%)
Transferrin index	14 (7.6%)	11 (6.0%)	26 (14.1%)	6 (3.3%)	127 (69.0%)
sTfR: SF ratio	5 (2.7%)	8 (4.3%)	33 (17.9%)	2 (1.1%)	136 (73.9%)
Body iron stores	4 (2.2%)	7 (3.8%)	34 (18.55)	2 (1.1%)	137 (74.5%)

*IDS* iron deficient stores, *SF* serum ferritin, *IDA* iron deficiency anaemia, sTfR serum transferrin ratio, *NIDA* non-iron deficiency anaemia, *CRP* C-reactive protein, *MA* anaemia with iron deficiency + inflammation, *NA* non-iron deficient non-anaemic

**Table 4:** Anaemia and iron status classification—comparison of the degree of agreement in categorising children’s anaemia status using measures of SF/CRP and using calculated body iron stores

	Iron status and anaemia		Agreement %	Kappa	p-value
	SF/CRP	Body iron stores			
IDS	3	4	75	0.6	0.01
IDA	6	7	85.7	0.78	0.00
NIDA	35	34	97.1	0.96	0.00
MA	2	2	100	1.00	0.00
Total	46	47			

*IDS* iron deficient stores*, SF* serum ferritin*, IDA* iron deficiency anaemia*, CRP* C-reactive protein, *NIDA* non-iron deficiency anaemia*, MA* anaemia with iron deficiency + inflammation

The use of SF/CRP and the sTfR: SF ratio correlated closely with the quantitative assessment of body iron stores. This was in contrast to when sTfR was used alone, as sTfR showed a wide variation in the interpretation of iron status.

## Discussion

Discrepancies were observed between SF/CRP, sTfR, TfR-Index sTfR/SF ratio and calculated body iron stores in the interpretation of iron status. The prevalence of IDA/NIDA differed depending on which index was used to measure iron status. The combination of sTfR and SF calculated as a ratio was a better solution in identifying iron status and differentiating between IDA and NIDA compared to the use of either sTfR or SF concentration alone for the clinical assessment of anaemia. Serum ferritin and CRP when used in combination showed the best agreement with body iron stores. The levels of sTfR were increased in the presence of inflammation and infections [9]. Thus in this current study sTfR levels were elevated in both IDA and NIDA. Hence as with SF, sTfR levels may not be particularly accurate in identifying iron deficiency when used as an individual assessment. Also of note, is that although sTfR levels were easily measured by the Elisa methods, lack of standardisation could result in large differences in the results obtained by different laboratories making it difficult to compare results. More research involving the concurrent determination of Hb, iron-status indices, inflammation status and other causes of anaemia in children is needed to add clarity to these inter-relationships. These factors need to be taken into consideration when identifying specific groups for targeted interventions and planning anaemia control programs in this region.

The presence of inflammation may have concealed some deficient iron levels [8]. Previous South African surveys have not measured inflammation, hence the contribution of anaemia associated with inflammation could not be compared. In this present study, CRP had a statistically significant relationship with ferritin. This observation is in keeping with iron sequestration during inflammation [8]. Markers of inflammation should therefore always be incorporated in studies assessing anaemia and iron status in similar environments. Hepcidin has been suggested as a possible indicator of iron status and the response to infection as it regulates iron levels and location in response to nutritional status and infection [23]. Hepcidin levels were not available in this low resource setting although its value in the assessment of iron status and the inflammatory process was acknowledged.

The determination of NIDA was a major difficulty, particularly because of the lack of suitable markers of infection or inflammation. A large number of anaemic children were classified as having NIDA because they could not be classified into IDA or MA groups. The cause of the anaemia in these children could not be verified at this time. Several researchers have assumed that the cause of relatively high SF levels in the presence of anaemia as observed in this study was due to inflammation or infection [9, 11, 12]. This deduction cannot be reinforced by our data due the lack of a suitable marker of infection and limitations of CRP. Our findings emphasize that nutritional iron deficiency should not be assumed to be the main contributor to anaemia in rural or even peri-urban settings of South Africa [24]. Precise measurement of iron status requires various parameters to be re-examined as wide variations were noted particularly with sTfR.

This study revealed a high anaemia prevalence of 43/184 (22.3%) and highlighted the importance of inflammation (79.1%) and iron deficiency (16.3%) in the aetiology of anaemia among children in this low-resource setting. The high anaemia prevalence reported in this present study was in contrast to the 2012 South African National Health and Nutrition Examination Survey (SANHANES-1) [25]. SANHANES-1 reported an anaemia prevalence (Hb < 110 g/l) of 10.5%, iron deficiency of 11% and IDA was identified among 2.1% of the children aged up to 14 years. Mean Hb was 122 g/l and mean ferritin was 40.8 μg/l. The researchers reported no statistically significant differences by locality or province. The results of the SANHANES-1 survey revealed improved anaemia and iron status for children in South Africa when compared to this current study and previous national surveys conducted in 1994 [26] and 2005 [27]. The age groups, Hb cut-off and indicators used for ID and IDA were not the same as those used for this current study. Bearing this in mind, the SANHANES-1 results are to be compared with caution. The anaemia prevalence reported in this research study was however consistent with earlier national surveys such as the, the South African Vitamin A Consultative Group (SAVACG) of 1994 as well as the 2005 National Food Consumption Survey-Fortification Baseline (NFCS-FB-I). These studies described an anaemia prevalence ranging from 21 to 33% for children aged up to 6 years, with anaemia being most prevalent in children aged 6–23 months [26–28]. In two separate studies in Kwazulu-Natal province, the prevalence of anaemia in school age children was 16.5 and 22% respectively [29, 30].

Differences in background co-morbidities were observed between anaemic and non-anaemic children. Inflammation and iron deficiency were more common among the anaemic children. A fair proportion (7.1%) of the children sampled were iron deficient but not anaemic. This was consistent with the 2013 SANHANES survey [26] which reported a national prevalence of 8.1% iron deficiency. Data from other national studies of children aged one to five years such as NFCS-FB-I [27] showed that about 14% had a poor iron status and SAVACG reported that iron deficiency was present in 10%, with IDA detected in 5% (Hb < 110 g/l and ferritin < 12 μg/l) [26]. The IDS group may have represented children who had been partially treated, were recovering from anaemia, and children with less severe or early iron depletion not yet sufficient to cause anaemia. Among children who had been previously treated for anaemia, the anaemia persisted in a large proportion. This could reflect inadequate initial treatments and follow-up or persistent causative factors. Children from this geographical location had a lower risk of iron deficiency than the national average [25]. The low prevalence of iron deficiency identified in this study was in contrast to previous surveys in the same province such as the 2001 study in UMkhanyakude and Zululand which reported that 33% of children aged 2–5 years were deficient in iron [30].

The contribution of parasite infestation to anaemia was statistically significant as also reported by previous studies in South Africa [29]. Some non-pathogenic organisms such as *E. coli* were strongly associated with anaemia, while some pathogenic organisms such as *G*. *lamblia* were weakly associated. This suggests that some of ‘non-pathogens’ are linked with anaemia and future research needs to re-examine the pathological significance of these organisms for specific conditions.

## Limitations

- Only locally accessible tests were used in the assessment of iron status and inflammation. This study did not measure inflammation in late convalescence. By omitting to measure chronic inflammation the categorisation of the causes of anaemia may have been biased. The ideal indicator of infection has not been documented yet, but preferably—would have a comparable reaction to infection as ferritin, not be affected by iron levels and be accessible in low resource settings. The acute-phase protein Orosomucoid (alpha-1-acid glycoprotein) whose serum concentration increases in response to systemic tissue injury, inflammation or infection [31], has been used for measuring late inflammation in resourced settings. However, like ferritin and CRP, orosomucoid responds differently in diverse conditions. Reduced orosomucoid levels have been described with intestinal infections while increased levels were reported for conditions such as burns, some drugs, pregnancy and certain diseases such as HIV [32]. The hepcidin anti-microbial peptide hormone which regulates iron levels and location in response to nutritional status and infection has also been recommended as an indicator of iron status and the response to infection [33]. Hepcidin controls iron availability and distribution by regulating dietary iron absorption as well as the movement of dietary, recycled and stored iron in body tissues [33]. Both orosomucoid and hepcidin tests were however not available in this low resource study setting.
- The reliability of the caregiver reports for measuring previous anaemia treatment and deworming treatments received by the children could not be established.
- None of the sampled children were known to have any hemoglobinopathies. The children enrolled in this study were all of black South African ethnicity. Compared to other parts of Africa, South Africa has a very low incidence of hemoglobinopathies. This demographic could however be changing due to immigration.
- The testing of one sample of stool/urine for *S. haematobium* infection was of limited sensitivity, particularly for children with light-intensity infections as low amounts of eggs are shed intermittently. The *Schistosoma* antigen test was not accessible in this study location.
- The details of accuracy and precision of the laboratory measurements done were not available.

## Conclusion

The findings of this study emphasize the need for population-based screening, and for epidemiologic findings to be incorporated into basic treatment algorithms for use during individual level care and for population based interventions. The determination of Hb concentration alone was considered to be inadequate. Identifying the cause of the anaemia using available resources was similarly important bearing in mind that the nature of anaemia varies according to the population and setting. The importance of measuring the contribution of iron deficiency to the population’s anaemia status, was particularly highlighted for settings where numerous contributory factors are expected to exist. The use of combined serum ferritin and transferrin receptors tests to identify the cause of anaemia, lessened the risk of incorrect diagnosis of iron deficiency and gave a more consistent index of body iron stores than that obtained from individual tests.

We reported very high anaemia prevalence in school-aged children from this sample population. This high burden of anaemia signals the need for a comprehensive methodology to its suppression. The anaemic children identified in this study were all asymptomatic. Given the slow and often asymptomatic character of anaemia and iron deficiency, greater prominence of protective actions and follow-up is required. The school health education and immunisation system are recommended as appropriate tools for increasing consciousness, and implementing effective interventions for anaemia control in school-age children.

The sTfR level which is an early marker of functional iron deficiency is widely believed to be unaffected by inflammation. Our study however supports evidence from other researchers who have reported that sTfR levels may be affected by the presence of inflammation [9]. Although a significant association was reported between sTfR and CRP, the sTfR was not adjusted for the presence of raised CRP because CRP was considered to be a non-specific indicator of conditions that result in raised sTfR levels even when iron stores are adequate. Nonetheless, the presence of inflammation may have had an impact on the estimated prevalence of iron deficiency using sTfR concentrations. Also of note is that the Cook model does not differentiate whether ‘normal iron stores’ are a result of sufficient dietary iron or concurrent inflammation which comes with high ferritin levels.
